# Band gap bowing in Ni_x_Mg_1−x_O

**DOI:** 10.1038/srep31230

**Published:** 2016-08-09

**Authors:** Christian A. Niedermeier, Mikael Råsander, Sneha Rhode, Vyacheslav Kachkanov, Bin Zou, Neil Alford, Michelle A. Moram

**Affiliations:** 1 Department of Materials, Imperial College London, Exhibition Road, London, SW7 2AZ, UK; 2 Diamond Light Source Ltd, Diamond House, Chilton, Didcot, Oxfordshire, OX11 0DE, UK

## Abstract

Epitaxial transparent oxide Ni_x_Mg_1−x_O (0 ≤ x ≤ 1) thin films were grown on MgO(100) substrates by pulsed laser deposition. High-resolution synchrotron X-ray diffraction and high-resolution transmission electron microscopy analysis indicate that the thin films are compositionally and structurally homogeneous, forming a completely miscible solid solution. Nevertheless, the composition dependence of the Ni_x_Mg_1−x_O optical band gap shows a strong non-parabolic bowing with a discontinuity at dilute NiO concentrations of *x* < 0.037. Density functional calculations of the Ni_x_Mg_1−x_O band structure and the density of states demonstrate that deep Ni 3d levels are introduced into the MgO band gap, which significantly reduce the fundamental gap as confirmed by optical absorption spectra. These states broaden into a Ni 3d-derived conduction band for *x* > 0.074 and account for the anomalously large band gap narrowing in the Ni_x_Mg_1−x_O solid solution system.

The wide-band gap semiconductor NiO (3.7 eV) is one of the few p-type transparent conductive oxides demonstrating good electrical properties for application in optoelectronic devices^1^. Thus NiO is used as a transparent hole transport layer in oxide p-n heterojunction devices such as electrical current rectifiers^2^,^3^, ultraviolet (UV) photodetectors^4^,^5^ and light-emitting diodes^6^,^7^. The epitaxial growth of high-crystal quality NiO thin films is desired, since polycrystalline films exhibit inferior electrical transport properties and may lead to higher leakage currents in p-n heterojunction devices.

The epitaxial growth of NiO on MgO single crystal substrates offers a promising platform for the preparation of complete UV-transparent oxide devices. Both NiO and MgO crystallize in the cubic rock-salt structure and Ni_x_Mg_1−x_O thin films over the entire composition range can be grown with high crystalline quality on MgO single crystals^8^, having a structural mismatch of only 0.8% at maximum^9^. As compared to many other semiconductor alloys, the Ni_x_Mg_1−x_O system is unique in demonstrating a great flexibility for band gap tuning from 3.7 eV to 7.8 eV^10^ in the deep UV region without the drawbacks of a significant change in lattice parameter or a phase transition. Therefore, Ni_x_Mg_1−x_O is increasingly receiving interest for application in deep UV photodetectors^8^,^11^,^12^,^13^,^14^. To tune the photosensitivity of these devices, it is essential to understand the Ni_x_Mg_1−x_O band gap evolution as a function of composition and to be able to accurately describe it using an analytical equation.

Experimentally it has been shown that the band gap dependence in most semiconductor alloys or solid solutions A_x_B_1−x_C as a function of composition *x* follows the parabolic function^15^


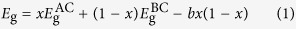


where 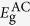 and 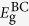 are the band gaps of the pure compounds AC and BC, respectively, and *b* is the bowing parameter. Since the band gap of the semiconductor alloy is generally smaller than indicated by the linear interpolation between the band gaps of its pure end members, the bowing parameter *b* is a positive constant. It has been proposed that the band gap bowing results from the aperiodic variation of the crystal potential in substitutional alloys, arising from random variations in occupation of the metal sites in the alloy by elements A and B, and its magnitude is a symmetric function of composition proportional to *x*(1 − *x*)^16^.

The Ni_x_Mg_1−x_O band gap dependence has been determined by optical absorption spectra of epitaxial thin films on MgO substrates prepared by molecular beam epitaxy^17^, textured thin films on quartz substrates prepared by magnetron sputtering^14^,^18^ and sol-gel spin coated thin films on quartz substrates^19^. Despite the large 7.8 eV MgO band gap, it has been observed that the Ni_x_Mg_1−x_O band gap increases only marginally to 4.8 eV, even at a small NiO fraction of only 8 at.%. A density functional theory (DFT) calculation of the Ni_x_Mg_1−x_O band gap dependence on composition has investigated superstructures with NiO contents of 25, 50 and 75 at.%^20^. However, since the study excludes the dilute NiO concentration regime for *x* < 0.25, no band gap discontinuity has been identified and the underlying Ni_x_Mg_1−x_O electronic band structure has not been investigated.

The present work combines experiment and theory to resolve the controversy over the Ni_x_Mg_1−x_O band gap bowing. Regardless of the strongly non-parabolic band gap dependence on composition, the standard bowing theory has been rigidly applied in previous studies to describe the band gap evolution in the Ni_x_Mg_1−x_O system^11^,^12^,^14^,^18^,^20^,^21^. Through a detailed investigation of the Ni_x_Mg_1−x_O microstructure and the underlying Ni_x_Mg_1−x_O electronic structure with a particular focus on the dilute NiO concentration regime (*x* = 0.125, 0.074 and 0.037), the present work demonstrates that I) the origin of the irregular Ni_x_Mg_1−x_O band gap dependence shall not be attributed to apparent structural or compositional inhomogeneities in Ni_x_Mg_1−x_O films and II) the standard bowing theory is inapplicable to describe the non-parabolic composition dependence of the Ni_x_Mg_1−x_O band gap because deep Ni 3d gap states evoke a highly anomalous bowing trend and significantly reduce the Ni_x_Mg_1−x_O band gap even at small NiO fractions of only 3.7 at.%.

## Results

### Structural characterization of Ni_x_Mg_1−x_O thin films

**Figure 1:**
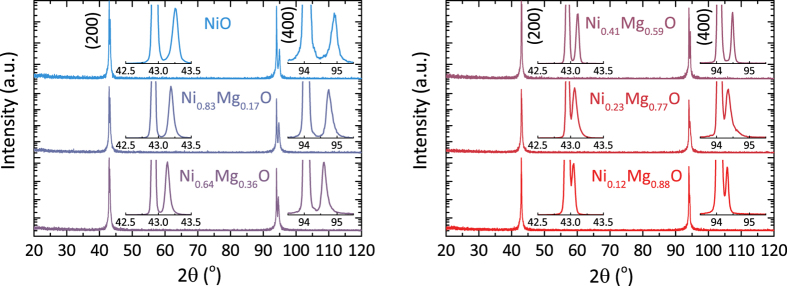
XRD *ω* − 2*θ* patterns of Ni_x_Mg_1−x_O thin films (*x* = 1, 0.83, 0.64, 0.41, 0.23, 0.12) prepared by pulsed laser deposition (PLD) showing the 100-oriented epitaxial growth on MgO(100) substrates. Insets show a magnification of the Ni_x_Mg_1−x_O 200 and 400 diffraction peaks on a linear scale.

In the *ω* − 2*θ* high resolution X-ray diffraction (HRXRD) patterns of the Ni_x_Mg_1−x_O thin films (*x* = 1, 0.83, 0.64, 0.41, 0.23, 0.12) only the 200 and 400 diffraction peaks are observed, indicating that films are single-phase and grown in 100-orientation on the MgO(100) substrates (Fig. 1). Due to the marginal difference in lattice parameters between NiO (4.177 Å) and MgO (4.212 Å)^9^, the Ni_x_Mg_1−x_O 200 and 400 diffraction peaks overlap with the corresponding peaks of the MgO substrate, especially for the Ni_x_Mg_1−x_O specimens of high MgO content. The insets show a clear shift of the Ni_x_Mg_1−x_O 200 and 400 diffraction peaks with increasing MgO fraction towards the MgO substrate peaks, indicating an increase in the out-of-plane lattice parameter.

**Figure 2:**
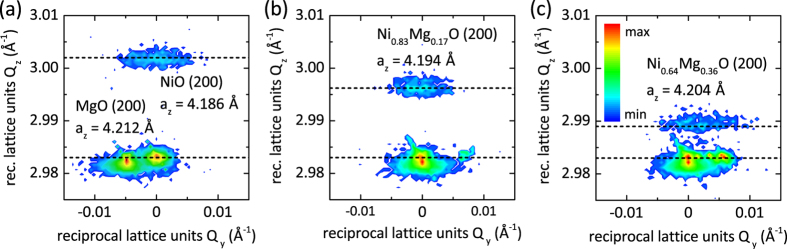
Projection of the 3D reciprocal space maps of the Ni_x_Mg_1−x_O 200 reflection along the reciprocal lattice vector *Q*_x_ of Ni_x_Mg_1−x_O thin films (x = 1, 0.83, 0.64) grown on MgO(100) substrates recorded with 6 keV (2.067 Å) synchrotron radiation. The intensity of the diffraction peaks is given by an iso-intensity contour map on a logarithmic scale. The dashed lines give the local intensity maxima of the Ni_x_Mg_1−x_O 200 and MgO 200 diffraction peaks as indicated.

The projection of the 3D reciprocal space maps of the Ni_x_Mg_1−x_O 200 reflection along the reciprocal lattice vector *Q*_x_ recorded with 6 keV synchrotron radiation is given by an iso-intensity contour map on a logarithmic scale (Fig. 2). The increased X-ray wavelength of 2.067 Å as compared to the Cu K_*α*1_ X-ray source of 1.5406 Å allows for a significantly improved spatial resolution of the high intensity 200 reflections of the Ni_x_Mg_1−x_O thin film and MgO substrate. Note that the observation of multiple peaks for the MgO 200 substrate indicates the presence of additional macroscopic crystallographic domains (twins). The relatively narrow Ni_x_Mg_1−x_O 200 diffraction peak in the in-plane *Q*_y_ direction in reciprocal space as compared to the MgO substrate indicates the high crystalline quality and low degree of mosaicity of the thin films. In particular, the out-of-plane width of the Ni_x_Mg_1−x_O 200 diffraction peaks along *Q*_z_ (*x* = 0.83, 0.64 in Fig. 2(b,c)) does not indicate any compositional broadening as compared to the pure NiO thin film (Fig. 2(a)). The Ni_x_Mg_1−x_O 200 diffraction peak shifts to smaller *Q*_z_ values with increasing MgO content in accordance with the slight increase in lattice parameter from NiO (4.177 Å) to MgO (4.212 Å). Reciprocal space map analysis of the asymmetric NiO 204 reflection shows that the in-plane lattice parameter *a*_*x*_ is larger than the unstrained reference (see Supplementary Fig. S1), indicating that the Ni_x_Mg_1−x_O thin films are not completely relaxed, but strained to match the in-plane MgO substrate lattice parameter.

**Figure 3:**
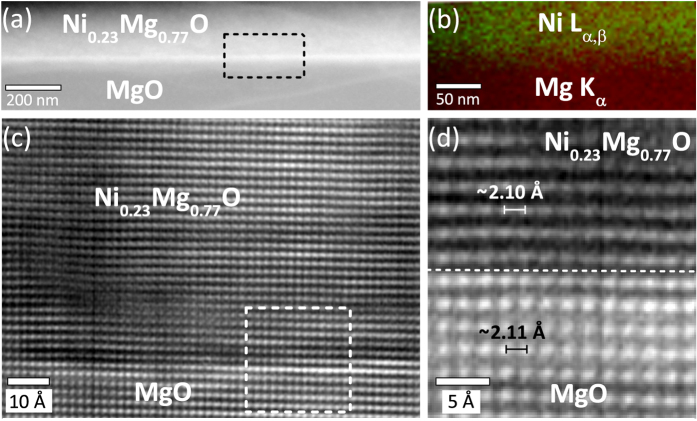
(**a**) Cross-sectional STEM-HAADF image of the Ni_0.23_Mg_0.77_O/MgO interface acquired along the <001> zone axis and (**b**) STEM-EDX elemental map showing the Ni L_α,β_ (green) and Mg K_α_ (red) emission intensity for the area marked with the box in (**a**). (**c**) Cross-sectional HRTEM image of the Ni_0.23_Mg_0.77_O/MgO interface acquired along the <001> zone axis demonstrating the single-domain epitaxial growth. (**d**) The high magnification ABSF HRTEM image of the region indicated with the box in (**c**) shows the coherent crystal interface between the Ni_0.23_Mg_0.77_O thin film and the MgO substrate due to the nearly identical lattice spacing parameter *d*_200_.

A cross-sectional scanning transmission electron microscopy high angle angular dark-field (STEM-HAADF) image of the Ni_0.23_Mg_0.77_O film on MgO substrate acquired along the <001> zone axis is shown in Fig. 3(a). To locate the Ni_0.23_Mg_0.77_/MgO interface, a STEM energy dispersive X-ray spectroscopy (EDX) elemental map employing the Ni L_α,β_ (green) and Mg K_α_ (red) emission lines was recorded (Fig. 3(b)). The cross-sectional high resolution transmission electron microscopy (HRTEM) image of the Ni_0.23_Mg_0.77_O/MgO interface acquired along the <001> zone axis using multi-beam conditions shows coherency and a defect-free Ni_0.23_Mg_0.77_O bulk, demonstrating the single-domain epitaxy of the film on the MgO substrate (Fig. 3(c)). The very close lattice matching and coherent crystal interface between the Ni_0.23_Mg_0.77_O film and MgO substrate is observed in the high magnification average background subtraction filtered (ABSF) HRTEM image (Fig. 3(d)) of the region indicated in Fig. 3(c).

### Optical transmission and band gap dependence

**Figure 4:**
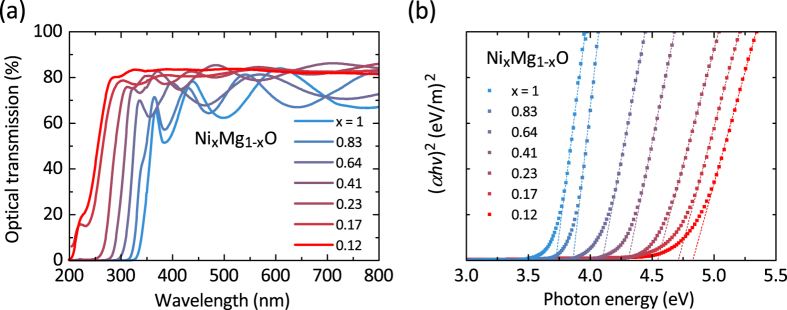
(**a**) Optical transmission of Ni_x_Mg_1−x_O thin films (*x* = 1, 0.83, 0.64, 0.41, 0.23, 0.17, 0.12) in the UV-visible range from 200 nm to 800 nm. (**b**) Optical absorption showing the linear relationship between the photon energy *hν* and (*αhν*)^2^, where *α* denotes the absorption coefficient, indicating a direct optical transition.

The optical transmission spectra of Ni_x_Mg_1−x_O thin films (*x* = 1, 0.83, 0.64, 0.41, 0.23, 0.17, 0.12) indicate about 80% transparency in the visible and UV region until the onset of their fundamental absorption (Fig. 4(a)). The optical absorption blue-shifts with increasing MgO content in the Ni_x_Mg_1−x_O thin films, from 340 nm for pure NiO to 200 nm for Ni_0.12_Mg_0.88_O. The calculated optical absorption spectra confirm the linear relationship between the photon energy *hν* and (*αhν*)^2^, where *α* denotes the absorption coefficient, which is valid for direct transitions in semiconductors at the absorption edge (Fig. 4(b))^22^.

**Figure 5:**
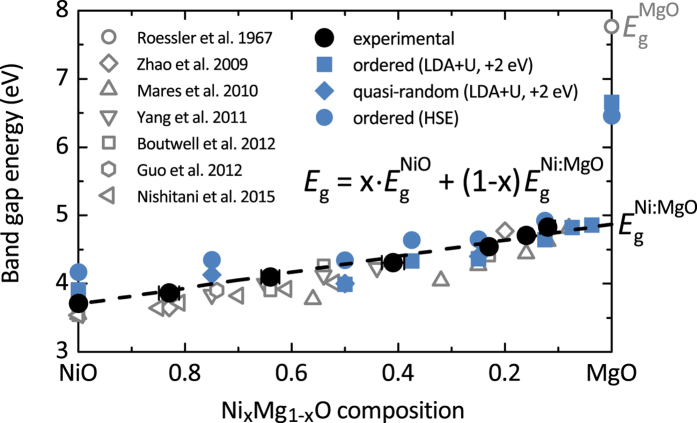
Experimental optical band gap of Ni_x_Mg_1−x_O thin films (*x* = 1, 0.83, 0.64, 0.41, 0.23, 0.17, 0.12) obtained by absorption spectra (black filled circles). The error in the determination of the optical band gap is contained within the size of the data points. Calculated electronic band gaps of Ni_x_Mg_1−x_O (*x* = 1, 0.75, 0.5, 0.375, 0.25, 0.125, 0.074, 0.037, 0) obtained from the DFT calculations of ordered (blue filled squares) and special quasi-random structures (blue filled diamonds), are shifted by +2 eV to account for the underestimation by the LDA + U method. In addition, the calculated electronic band gaps from the DFT calculation of ordered structures using the HSE approach are presented (blue filled circles). Experimental optical band gaps which have been determined by absorption and reflectance spectra in previous studies are included for comparison (grey empty symbols, after Roessler, D. M. *et al*.^10^, Zhao, Y. *et al*.^12^, Nishitani, H. *et al*.^14^, Mares, J. W. *et al*.^17^, Yang, Z.-G. *et al*.^18^, Boutwell, R. *et al*.^19^ and Guo, Y. M. *et al*.^23^).

The composition dependence of the Ni_x_Mg_1−x_O optical band gap as determined from the absorption spectra presented in this work is shown Fig. 5. The optical band gaps which have been determined in previous studies of Ni_x_Mg_1−x_O thin films prepared by electron beam evaporation^12^, molecular beam epitaxy^17^, magnetron sputtering^14^,^18^, pulsed laser deposition^23^ and sol-gel spin coating^19^ are included for comparison. In this work, the measured optical band gap of pure NiO is 3.7 eV and by alloying with MgO it increases linearly to 4.8 eV for Ni_0.12_Mg_0.88_O. Despite the small NiO fraction of only 12 at.%, it is striking to observe that the measured optical band gap remains 3 eV below that of pure MgO.

### Density functional theory calculation of the Ni_x_Mg_1−x_O electronic structure

The calculated electronic band gaps obtained from DFT calculations of both ordered and special quasi-random structures (SQS) of Ni_x_Mg_1−x_O are included in the band gap plot (Fig. 5). To account for the underestimation of the calculated band gaps for NiO (1.9 eV) and MgO (4.7 eV) applying the local density approximation (LDA + U) method, these values are shifted by +2 eV for comparison with experiment. The hybrid density functional calculations using the Heyd, Scuseria and Ernzerhof (HSE) approximation yields band gaps for NiO (4.2 eV) and MgO (6.5 eV) in good agreement with experiment. In consistence with the experimentally measured optical band gap trend, the calculations confirm that upon alloying with MgO, the Ni_x_Mg_1−x_O band gap increases linearly over the entire investigated composition range (1 ≤ *x* ≤ 0.037). Both LDA + U and HSE calculation methods demonstrate that the variation in the band gap between NiO and Ni_0.125_Mg_0.875_O is only about 0.7 eV. However, the difference between the calculated band gaps of Ni_0.037_Mg_0.963_O and pure MgO of 1.8 eV is surprisingly large. More than 65% of the total band gap variation in the Ni_x_Mg_1−x_O is observed after alloying just a few percent of NiO to MgO.

**Figure 6:**
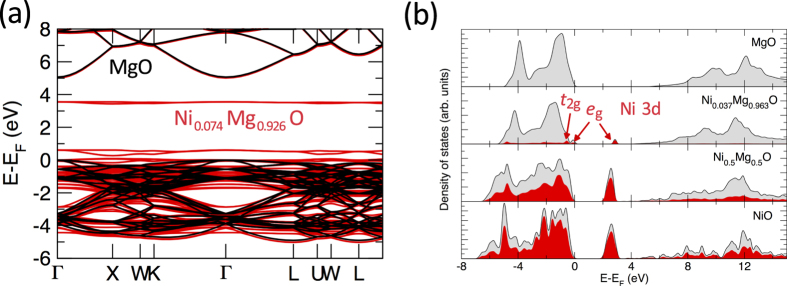
(**a**) Superposition of the electronic band structure of pure MgO (black) and Ni_0.074_Mg_0.926_O (red) obtained within the LDA + U method showing deep localized states derived from Ni 3d *e*_g_ states inside the MgO band gap. The Fermi level *E*_F_ of MgO is located at the valence band maximum and the Ni_0.074_Mg_0.926_O band structure is shifted to coincide with the MgO conduction band minimum. The Ni_0.074_Mg_0.926_O states at the top of the valence band are all occupied. (**b**) Calculated DOS of Ni_x_Mg_1−x_O (*x* = 1, 0.5, 0.037 and 0) indicating that the Ni 3d *e*_g_ states comprising the NiO conduction band remain as localized impurity states inside the MgO band gap for dilute NiO concentrations of 3.7 at.%. The partial DOS of Ni 3d states is shown in red.

A superposition of the calculated band structure of Ni_0.074_Mg_0.926_O and that of pure MgO obtained within the LDA + U method is shown in Fig. 6(a). It is striking to observe that the Ni_0.074_Mg_0.926_O band structure shows deep localized levels almost entirely derived from Ni 3d *e*_g_ states at about 3 eV inside the MgO band gap. The Ni 3d *e*_g_ impurity-like level remains localized as there is only a negligible contribution from hybridization with O 2p states of the surrounding atoms. Both the Ni_0.074_Mg_0.926_O valence and conduction band show a nearly unperturbed electronic structure derived from pure MgO. Since the extended states of the MgO conduction band are not affected by the localized Ni 3d *e*_g_ states, the approximation of employing a composition-independent +2 eV energy shift for the calculated band gaps obtained within the LDA + U method is justified to compare these results with experiment.

The calculated density of states (DOS) of Ni_x_Mg_1−x_O (*x* = 1, 0.5, 0.037 and 0) is shown in Fig. 6(b). A small concentration of 3.7 at.% NiO in MgO creates localized states inside the MgO band gap: I) the energy level derived from Ni 3d *e*_g_ and *t*_2g_ states just above the MgO valence band, which is occupied, and II) the energy level almost entirely derived from Ni 3d *e*_g_ states at about 3.5 eV. Both of these features show localized levels similar to those introduced by impurities, even though a concentration of 3.7 at.% NiO is considered in the alloying regime. With increasing NiO concentration, the lower energy Ni 3d *e*_g_ and *t*_2g_ levels merge with the O 2p states at the top of the valence band while the density of the higher energy Ni 3d *e*_g_ impurity level increases and broadens to form the Ni_0.5_Mg_0.5_O conduction band.

## Discussion

By alloying with MgO, the NiO band gap increases about linearly from 3.7 eV to 4.9 eV in Ni_0.037_Mg_0.963_O. For MgO doped with impurity concentrations below 3.7 at.% NiO a band gap discontinuity is expected. It is obvious that the standard bowing equation cannot be applied to describe the anomalous Ni_x_Mg_1−x_O band gap trend, because the bowing term *bx*(1 − *x*) only accounts for a parabolic deviation of the interpolated band gaps as a function of composition *x*, as a result of the aperiodic variation of the crystal potential in the random alloy.

The very narrow Ni_x_Mg_1−x_O 200 diffraction spots obtained by HRXRD shown in Fig. 2 do not show any peak broadening in the out of plane *Q*_*z*_ component in reciprocal space indicating that compositional fluctuations on a macroscopic level are insignificant. Compositional inhomogeneities are further very unlikely to be observed as it has been shown experimentally that NiO and MgO are fully miscible, typically forming a homogeneous Ni_x_Mg_1−x_O solid solution^24^,^25^. A calorimetric investigation as well as thermodynamic modelling of the NiO-MgO system report a negative enthalpy of mixing describing a tendency towards atomic ordering rather than clustering^26^,^27^. The microstructural homogeneity and high-quality epitaxial growth of the Ni_x_Mg_1−x_O is evident from the HRTEM images showing a defect-free Ni_x_Mg_1−x_O/MgO interface and suggesting single-domain epitaxial growth without evidence of either ordering or clustering (cf. Fig. 3).

The Ni_x_Mg_1−x_O band gap bowing cannot be attributed to any transition in crystal structure, direct-to-indirect electronic transition or variation in compositional homogeneity. However, calculations of the electronic properties of semiconductor alloy systems have previously shown that the band gap bowing coefficient may nevertheless become largely composition dependent if the dilute alloy shows a localized deep impurity level in the band gap^28^. When there is a pronounced difference in properties between the alloy end members, the impurities in the dilute alloy may create new electronic states rather than altering the host energy levels^29^.

Electronic structure calculations of 3d transition metal (Fe, Co, Ni) impurities in MgO reveal that deep energy levels in the band gap are formed by combination of the metal 3d orbitals with O 2p orbitals to form *e*_g_ and *t*_2g_ states^30^. Absorption spectra, cathodoluminescence and photoluminescence measurements of Ni-doped MgO crystals further indicate electronic transitions attributed to the energy levels of Ni^2+^ impurities^31^,^32^,^33^. The DFT calculations presented in this work demonstrate that even at dilute NiO concentrations of 3.7 at.%, well beyond typical defect concentrations, there is only a small hybridization between the Ni 3d and MgO extended states, creating such localized impurity-like levels inside the band gap. With increasing NiO content, the localized Ni 3d *e*_g_ states broaden to form the Ni_x_Mg_1−x_O conduction band. The anomalous Ni_x_Mg_1−x_O band gap narrowing is thus attributed to the fundamental difference in electronic structure between NiO and MgO, resulting in a remarkable modification of the MgO host band structure upon alloying with only a dilute amount of NiO (*x* ≤ 0.037).

The origin of the Ni_x_Mg_1−x_O band gap bowing is fundamentally different from that of most substitutional semiconductor alloys, for which the standard bowing equation holds. Instead, the non-parabolic Ni_x_Mg_1−x_O bowing behaviour induced by the Ni 3d-derived localized states can be related to the band gap narrowing observed for highly mismatched III-V semiconductor alloys such as GaN_x_As_1−x_^34^ and II-VI semiconductor alloys such as ZnS_x_Te_1−x_^35^. Alloying of GaAs (ZnTe) with a small amount of N (S) introduces localized states at an energy level close to the conduction band edge, resulting in splitting of the conduction band into subbands and an effective narrowing of the fundamental gap^36^,^37^. However, the Ni_x_Mg_1−x_O solid solution system is different in that the localized Ni 3d e_g_ states remain as localized impurity states well inside the MgO band gap while no perturbation of the host conduction band structure is observed (cf. Fig. 6(a)). The Ni_x_Mg_1−x_O solid solution presents a unique system in which cation substitution of a small percentage of Ni^2+^ for Mg^2+^ leads to a fundamental change in the electronic structure, while maintaining complete miscibility as well as structural stability over the entire composition range.

The Ni_x_Mg_1−x_O band gap trend is best described by a linear interpolation of the NiO band gap 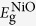 (3.7 eV) and that of MgO doped with an infinitesimal Ni impurity concentration 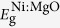 (4.9 eV):


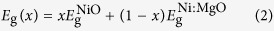


The results presented in this work show a critical concentration of about 7.4 at.% NiO in Ni_x_Mg_1−x_O beyond which the defect-like impurity states become delocalized through the interaction of neighbouring Ni^2+^ cations distributed on the cation sublattice of the cubic rock-salt structure. For a random distribution of Ni^2+^ and Mg^2+^ cations on the face-centered cubic sublattice of Ni_x_Mg_1−x_O, the calculated site percolation threshold of nearest-neighbour Ni^2+^ cations is 19.9 at.% NiO^38^. The observed threshold at about 7.4 at.% NiO defines a “localized-to-delocalized” transition (cf. ref. 29) at which the Ni_x_Mg_1−x_O electronic structure changes from the characteristics of a concentrated Ni_x_Mg_1−x_O solid solution with interacting Ni^2+^ cations to that of a dilute Ni:MgO solid solution with isolated Ni^2+^ impurity centres creating localized states in the MgO band gap. This indicates that the length scale over which Ni^2+^-Ni^2+^ interactions can appear is greater than predicted assuming only nearest-neighbour interactions, or the non-random ordering of Ni^2+^ atoms on the cation sublattice sites decreases the probability of Ni^2+^ nearest-neighbour site occupation.

## Conclusion

Despite forming a completely miscible and compositionally homogeneous solid solution, alloys of NiO and MgO exhibit a strikingly anomalous band gap bowing behaviour. The non-parabolic Ni_x_Mg_1−x_O band gap dependence is attributed to the fundamental difference in electronic structure between NiO and MgO, resulting in a remarkable modification of the MgO host band structure upon alloying with only a dilute amount of NiO (*x* ≤ 0.037). DFT calculations of the Ni_x_Mg_1−x_O band structure and density of states demonstrate that localized Ni 3d impurity levels are introduced at an energy well below the MgO conduction band and account for the pronounced band gap narrowing as confirmed by optical absorption spectra. The standard bowing theory is inapplicable to describe the Ni_x_Mg_1−x_O band gap dependence and may in general not be applied to semiconductor systems, for which one of the alloy end members creates such deep levels in the band gap.

## Methods

Single-phase polycrystalline Ni_x_Mg_1−x_O ceramic targets were prepared by sintering high purity NiO (99.999%) and MgO (99.995%) powders at temperatures of 1200–1550 °C for 5 h, applying higher temperatures for the targets of high MgO content. Ni_x_Mg_1−x_O thin films were grown on single-crystal MgO (100) substrates by pulsed laser ablation of the Ni_x_Mg_1−x_O ceramic targets using a 248 nm KrF excimer laser. The growth temperature was 600 °C and the deposition pressure was 0.7 Pa O_2_. The Ni_x_Mg_1−x_O ceramic targets were ablated with a laser beam fluency of 0.8 J/cm^2^ per pulse at a frequency of 10 Hz, resulting in a film thickness of about 300 nm.

The composition of the Ni_x_Mg_1−x_O thin films was accurately determined by EDX in a JEOL JSM-6010LA scanning electron microscope (SEM). Using a primary beam voltage of 5 kV, EDX spectra of the Ni L_α_ (852 eV), Ni L_β_ (869 eV) and Mg K_α_ (1254 eV) X-ray emission lines were recorded. The background-corrected, integrated peaks of the Ni L_α_, Ni L_β_ and the Mg K_α_ emission for all Ni_x_Mg_1−x_O thin films were evaluated based on a calibration curve obtained using data from the polycrystalline Ni_x_Mg_1−x_O targets of known composition. It was confirmed that the 5 kV primary electron beam is entirely probing the Ni_x_Mg_1−x_O thin films and does not reach the MgO substrate by investigating Ni_x_Mg_1−x_O thin films of equal layer thickness grown onto Si substrates for which the Si K_α_ emission (1740 eV) was not observed.

The crystal structure of the Ni_x_Mg_1−x_O thin films on MgO substrates was studied by HRXRD employing a Philips PANalytical MRD diffractometer equipped with a Cu-K_*α*1_ X-ray source (1.5406 Å) utilizing parallel beam geometry. The structure and crystalline quality of the Ni_x_Mg_1−x_O thin films on MgO were further investigated by HRXRD carried out on beamline B16 at the Diamond Light Source, UK, using a 6 keV (2.067 Å) monochromated X-ray source. The area detector was fixed at the expected 2*θ* value of the Ni_x_Mg_1−x_O 200 diffraction peak while scanning the incidence angle *ω* to record 2-dimensional *δ*-*χ* diffraction patterns, where *δ* is the angle between the detector arm and the horizontal plane and *χ* is the angle between the detector arm and the vertical plane. 3-dimensional (3D) reciprocal space maps were calculated from the obtained data set which show the Ni_x_Mg_1−x_O 200 diffraction peak intensity as a function of the components of the scattering vector *Q*_x_, *Q*_y_ and *Q*_z_ in reciprocal space. In this notation, *Q*_x_ and *Q*_y_ represent two orthogonal in-plane components and *Q*_z_ represents the out-of-plane component of the scattering vector in the specimen frame of reference.

The electron transparent cross-sectional TEM specimen was prepared by grinding, polishing and dimpling until the specimen thickness was below 10 μm, followed by Ar ion milling using a PIPS Ion miller (Gatan USA). Conventional HRTEM and STEM using the HAADF detector and STEM-EDX was performed using a JEOL 2100 microscope equipped with a field emission gun operating at 200 keV. The high magnification HRTEM image was filtered using an ABSF. STEM-HAADF and STEM-EDX studies were used to identify the Ni_x_Mg_1−x_O/MgO interface. EDX spectral images were acquired recording Ni L_α_, Ni L_β_ and Mg K_α_ X-ray emission lines and were analysed using the INCA software (ETAS group). The optical transmission of the Ni_x_Mg_1−x_O thin films were measured with a Cary 5000 UV-Vis-NIR spectrophotometer using a bare MgO substrate as 100% transmission reference.

To describe the Ni_x_Mg_1−x_O band gap trend, DFT calculations were performed using the LDA + U approach of Dudarev *et al*.^39^ and the hybrid density functional HSE approximation^40^,^41^. The LDA + U method was chosen in order to accurately describe the Ni 3d states because it significantly improves the performance of DFT applied to systems containing localised d electrons. The projector augmented wave method^42^ as implemented in the Vienna ab initio simulation package (VASP) was applied^43^,^44^. A plane wave energy cut-off of 800 eV was used in the calculations and the minimum k-point density is 0.4 Å^−1^. For all Ni_x_Mg_1−x_O calculations, the Coulomb and exchange energy parameters, *U* = 4.3 eV and *J* = 1.0 eV, were applied to the Ni 3d states according to previous studies^45^. It is noted that the Dudarev approach to LDA + U only depends on an effective energy parameter *U*_eff_ = *U* − *J* of 3.3 eV. The HSE approximation was chosen since it provides more accurate band gap values for semiconductors and insulators compared to traditional density functional approximations. In the HSE approximation the usual Hartree-Fock exchange mixing coefficient of 0.25 was used and the range separation parameter was set to 0.2 Å^−1^.

NiO is an antiferromagnet (AF), in which different Ni layers along the [111] direction have magnetic moments pointing in opposite directions. Thus magnetic moments on the Ni atoms in Ni_x_Mg_1−x_O may order antiferromagnetically and therefore calculations on Ni_x_Mg_1−x_O were performed based on the rock-salt derived structure of NiO with AF ordered layers of Ni atoms along the [111] direction (cf. refs 20 and 46). In the case of Ni_0.074_Mg_0.926_O, a 3 × 3 × 3 repetition of the primitive rock-salt structure was used in which two Ni atoms substitute for two Mg atoms, while maintaining the AF coupling of the Ni moments. To describe the unordered Ni_x_Mg_1−x_O solid solution, special quasi-random structures (SQS) were used^47^,^48^ and set up using the Alloy Theoretical Automated Toolkit (ATAT)^49^ for *x* = 0.75, 0.5 and 0.25. For all concentrations, the pair correlation functions for the first four atomic shells matched exactly with the pair correlation functions of the ideal unordered Ni_x_Mg_1−x_O solid solution. Using this method the obtained structures contained a total of 64, 32 and 64 atoms for *x* = 0.75, 0.5 and 0.25, respectively. In addition to the SQS model structures, calculations were performed using ordered supercells based on 2 × 2 × 2 and 3 × 3 × 3 repetitions of the rock-salt derived AF NiO structure. Both approaches were applied to investigate the influence of atomic ordering on the calculation results. The lattice constants of the Ni_x_Mg_1−x_O solid solution were determined from the experimentally measured lattice constants of NiO (4.177 Å) and MgO (4.212 Å) according to Vegard’s law^9^.

It shall be noted that other approaches such as the Green’s function based methods employing the coherent potential approximation (CPA) can provide an accurate description of random compound alloys^50^. The CPA is an effective method in describing the band structures of III-V alloys when disorder determines the band gap bowing^51^,^52^, and in particular for calculation of detailed band properties such as effective masses and absorption line broadening^53^. However, the single-site CPA does not account for local environment effects which can be important to describe semiconductors and require cluster expansion methods^54^. The supercells used in this study are only idealized descriptions of the random system, since the structures are periodically repeated in space and therefore have a translational symmetry in contrast to the real random alloy. Therefore, certain effects found in real disordered systems, such as transition energy broadening due to disorder and corresponding lifetime effects are neglected in the supercell methods. Even so, for this study, SQS in combination with ordered supercells are employed which closely reproduce the physically most relevant correlation functions of the infinite, random alloy and thus provide an accurate description of the band gap bowing^48^,^55^, while benefiting from a limited system size and reasonable computational effort.

## Additional Information

**How to cite this article**: Niedermeier, C. A. *et al*. Band gap bowing in Ni_x_Mg_1−x_O. *Sci. Rep.* **6**, 31230; doi: 10.1038/srep31230 (2016).

## Supplementary Material

Supplementary Information
