# Supplementary material for: Band gap bowing in NixMg1−xO
Source: Sci Rep. 2016 Aug 9;6:31230. doi: 10.1038/srep31230 (PMC4977526; doi:10.1038/srep31230)
Supplement: Supplementary Information [file srep31230-s1.pdf]

Supplementary Material: Band gap bowing in $Ni_xMg_{1-x}O$

Christian A. Niedermeier[1,*], Mikael Råsander[1], Sneha Rhode[1], Vyacheslav Kachkanov[2,†], Bin Zou[1], Neil Alford[1], Michelle A. Moram[1]

[1]Department of Materials, Imperial College London, Exhibition Road, London, SW7 2AZ, UK
[2]Diamond Light Source Ltd, Diamond House, Chilton, Didcot, Oxfordshire, OX11 0DE, UK
[†]present address: Tokamak Energy Ltd, 120A Olympic Avenue, Milton Park, Oxfordshire OX14 4SA, UK
[*]c.niedermeier13@imperial.ac.uk

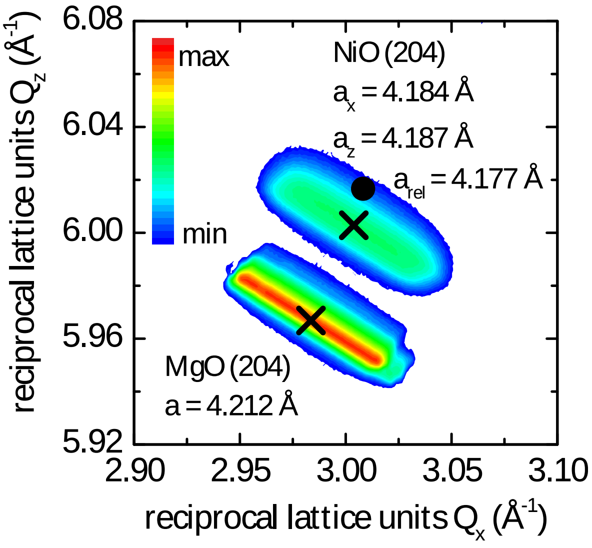

Figure S1. Reciprocal space map of the NiO 204 reflection recorded with Cu $K_{\alpha 1}$ radiation (1.5406 Å). The intensity of the diffraction peaks is given by an iso-intensity contour map on a logarithmic scale.
